# Work Characteristics and Occupational Well-Being: The Role of Age

**DOI:** 10.3389/fpsyg.2016.01411

**Published:** 2016-09-22

**Authors:** Hannes Zacher, Antje Schmitt

**Affiliations:** ^1^School of Management, Queensland University of TechnologyBrisbane, QLD, Australia; ^2^Department of Business Psychology, Economics and Management, University of KasselKassel, Germany; ^3^Institute of Psychology, University of KasselKassel, Germany

**Keywords:** age, aging, lifespan, work characteristics, well-being

## Abstract

Based on a lifespan perspective on work design, person-environment interaction and fit theories, models of successful aging at work, and role theory, we review research on the role of worker age in relationships between work characteristics and occupational well-being. We first focus on interaction effects of work characteristics and age on occupational well-being. Research has found that age can moderate associations between work characteristics and occupational well-being indicators, and that work characteristics can moderate associations between age and occupational well-being indicators. Next, we describe research showing that work characteristics can mediate associations between age and occupational well-being indicators. The relationships of age with specific work characteristics and occupational well-being indicators can be linear or non-linear. We conclude our literature review by discussing implications for future research.

## Introduction

Due to rapid population and workforce aging in many countries, organizational researchers and practitioners have become increasingly interested in the role of age in the work context (Finkelstein et al., 2015; Truxillo et al., 2015). In this article, we review research in one particular area within the growing field of work and aging: the role of age in relationships between work characteristics and occupational well-being. Research in this area is important because work characteristics and work (re)design can have differential effects on younger and older workers' well-being (Griffiths, 1999; Truxillo and Zaniboni, 2015) and may influence how workers' well-being develops across their careers (Matthews, 2015; Schmitt and Bathen, in press).

The vast majority of studies on work characteristics and occupational well-being has not adopted a lifespan perspective or considered age as a substantial variable. However, the number of studies on work characteristics and occupational well-being that have considered the role of age has increased over the past decade (Hertel and Zacher, in press). Consistent with the lifespan developmental literature (Baltes, 1987), we conceive age as a continuous variable and use the labels “older workers” and “younger workers” for descriptive purposes to refer to relatively higher and lower values of age, respectively. As our focus is on workers, the typical age range in studies on age, work characteristics, and occupational well-being is 18–65 years, with some variation at each end of the age continuum. While no generally accepted cut-off exists, for practical purposes, most organizations define “older workers” as those individuals either 40, 45, or 50 years and older (Kooij et al., 2008). With regard to occupational well-being, we adopt a broad definition that includes both subjective and objective indicators of physical, mental, and social well-being in the work context (World Health Organization, 2004; Schmitt, in press). This definition also includes both positive (e.g., good physical health, job satisfaction) and negative (e.g., ill-health, strain, emotional exhaustion) aspects of occupational well-being.

**Figure 1:**
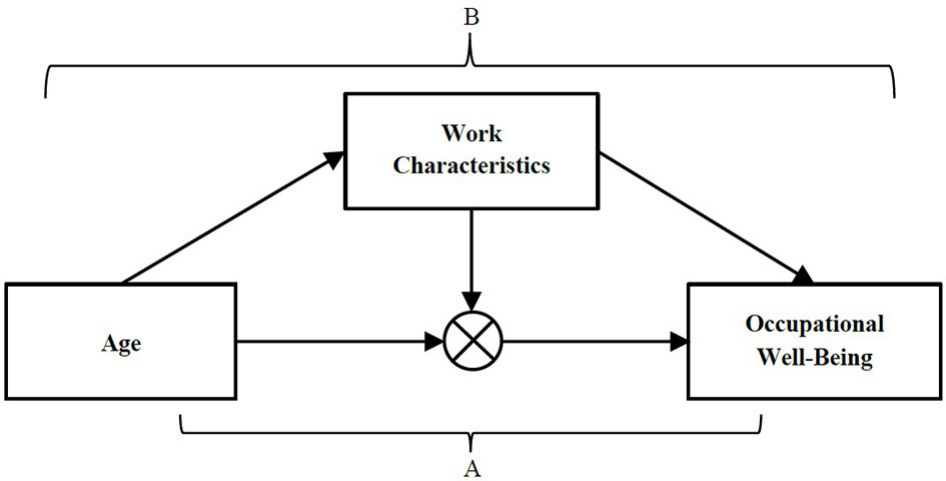
**Conceptual Framework of Relationships among Age, Work Characteristics, and Occupational Well-Being**. Pathway A represents the interaction effect of age and work characteristics on occupational well-being. Pathway B illustrates the role of work characteristics as mediators of the association between age and occupational well-being.

In the following sections, we first review four important theoretical frameworks (i.e., a lifespan perspective on work design, person-environment interaction and fit theories, models of successful aging at work, and role theory) that can help explain the role of age in relationships between work characteristics and occupational well-being. Second, we report the methods and results of our literature review on age, work characteristics, and occupational well-being. This literature review is structured based on the conceptual framework shown in Figure 1. On the one hand, we review research on interaction effects of work characteristics and age on occupational well-being (Pathway A in Figure 1). Interaction effects indicate that the relationship between two variables depends on the level of a third or moderator variable (Fairchild and MacKinnon, 2009). The moderator variable may affect the direction and/or strength of the relationship between the other two variables. We focus on work characteristics as moderators of associations between age and occupational well-being indicators, and on age as a moderator of associations between work characteristics and occupational well-being indicators. On the other hand, we review research on work characteristics as mediators of associations between age and occupational well-being indicators (Pathway B in Figure 1). A mediator variable connects a predictor with an outcome variable and may explain why the predictor is related to the outcome variable (Fairchild and MacKinnon, 2009). We conclude our review by outlining implications for future research.

## Theoretical background

The theoretical frameworks outlined in this section are useful to explain the possible relationships among age, work characteristics, and occupational well-being depicted in Figure 1. The lifespan perspective on work design, person-environment interaction and fit theories, as well as models of successful aging at work are primarily used to develop theoretical rationales for hypotheses on interaction effects of work characteristics and age on occupational well-being. More specifically, differential associations of work characteristics with occupational well-being among younger and older workers can be explained by the lifespan perspective on work design and person-environment interaction models. In contrast, work characteristics as moderators of associations between age and occupational well-being can be explained by person-environment fit models and models of successful aging at work. Finally, role theory has been used primarily to explain why work characteristics may mediate associations between age and occupational well-being indicators.

### Lifespan perspective on work design

Truxillo et al. (2012) combined lifespan and work design theories, such as socioemotional selectivity theory (Carstensen et al., 1999), the model of selection, optimization, and compensation (Baltes and Baltes, 1990), and job characteristics theory (Hackman and Oldham, 1976) into a comprehensive lifespan perspective on work design. They proposed that six task, knowledge, and social work characteristics (i.e., job autonomy, task significance, skill variety, specialization, social support, and interdependence; see Morgeson and Humphrey, 2006) are more strongly positively related to indicators of occupational well-being (i.e., job satisfaction, engagement) among older workers. In contrast, they suggested that task variety, feedback, interaction outside the organization, and receiving and providing feedback are more strongly positively related to occupational well-being among younger workers. For job complexity, information-processing demands, and problem-solving demands, Truxillo et al. (2012) suggested that the effects of these work characteristics among younger and older workers depend on their specific nature, that is, whether they primarily require fast information processing abilities (which tend to decrease with age) or experiential knowledge (which tends to be stable or increase with age; Kanfer and Ackerman, 2004). The authors further argued that the interaction effects of age, age-related person characteristics (e.g., cognition, personality, time perspective, experience), and work characteristics on occupational well-being are mediated by experienced meaningfulness of work, responsibility, and knowledge of results (cf. Hackman and Oldham, 1976), perceived person-environment fit, and motivation.

### Person-environment interaction and fit theories

In their lifespan perspective on emotion regulation, stress, and well-being, Scheibe and Zacher (2013) integrated the transactional model of stress (Lazarus and Folkman, 1984) and affective events theory (Weiss and Cropanzano, 1996) with the emotional aging literature (Charles and Carstensen, 2010). They proposed that age and age-related factors (e.g., emotional competencies, appraisal processes, changes in life contexts) interact with work characteristics and stressful work events in predicting occupational well-being. In a subsequent paper, Zacher et al. (2014a) integrated the person-environment fit approach to occupational well-being (Edwards et al., 1998) with the lifespan developmental literature (Baltes, 1987) to explain how interactions between age-related changes in person factor (i.e., traits, abilities, and needs) and age-related changes in contextual factors (i.e., other people, work demands, and supplies) can influence occupational well-being (see also Feldman and Vogel, 2009; Perry et al., 2012). They argued that these effects are mediated by the objective and perceived demands-abilities and needs-supplies fit between a worker and his or her job, team, organizational, and occupational characteristics.

### Models of successful aging at work

Successful aging at work involves a process during which workers maintain or improve favorable work outcomes, such as motivation, performance, and well-being with increasing age (Kooij, 2015; Zacher, 2015). Based on the principle of “differential preservation” from the lifespan developmental literature (Salthouse, 2006), Zacher (2015) proposed that empirical research on successful aging at work needs to demonstrate evidence for interaction effects between age and individual resources (e.g., action regulation strategies) and/or contextual resources (e.g., work characteristics), such that resources explain more variance in work outcomes among older compared to younger workers. Older workers who experience relatively higher levels of job satisfaction and engagement compared to the average older worker can be said to have aged successfully in terms of occupational well-being.

### Role theory

While research in the lifespan developmental psychology tradition has focused on age-related changes in individual difference characteristics such as cognitive and physical abilities, personality, and motives (Baltes et al., 2006), age may also be associated with certain work characteristics which, in turn, relate to occupational well-being. A useful explanation for this mediating role of work characteristics in associations between age and occupational well-being is provided by role theory, which suggests that workers occupy multiple roles within and outside the work context (e.g., worker, colleague, family member), and that the perception and perceived importance of these roles, and more specific tasks, expectations, and available resources within these roles, change over time and with age (Biddle, 1986; Ashforth, 2001). For instance, researchers suggested that workers in mid-career experience greater work and family demands, more work-family conflict, and less social support at work than their younger and older colleagues (Huffman et al., 2013; Zacher et al., 2014b).

## Literature review

We first describe our literature search strategy, followed by reviews of studies on interaction effects of work characteristics and age on occupational well-being, and of studies on work characteristics as mediators of associations between age and occupational well-being. Table 1 summarizes findings on age, work characteristics, and occupational well-being.

**Table 1:** **Summary of Findings on Age, Work Characteristics, and Occupational Well-Being**.

**Role of work characteristics and age**	**Indicator of occupational well-being**	**Main findings and associated empirical studies**
**WORK CHARACTERISTICS AS MODERATORS**		
• Work arrangements	Job satisfaction	• Stronger positive relationship between age and job satisfaction among full-time workers compared to workers in other work arrangements (Riordan et al., 2003).
• Job autonomy	Strain	• Age-differential effects of avoidance coping strategies on job strain are moderated by job autonomy, such that younger workers use more avoidance coping strategies than older workers when job autonomy is low (Hertel et al., 2015).
• Job autonomy and job complexity	Future work opportunities	• Job autonomy and job complexity buffer the negative relationship between age and favorable perceptions of future work opportunities (Zacher and Frese, 2009, 2011; Zacher et al., 2010).
**AGE AS MODERATOR**		
	Job satisfaction	• Job autonomy and feedback are more strongly positively related to job satisfaction among older compared to younger workers (Bos et al., 2013; Zaniboni et al., 2016).
		• Older workers report lower job satisfaction than younger workers when experiencing a misfit between personal needs and work-related supplies (Krumm et al., 2013).
		• Older workers' job satisfaction is more negatively affected than that of younger workers when they experience high levels of job insecurity (Mauno et al., 2013).
		• Younger workers' job satisfaction is more negatively affected than that of older workers by high workload and perceived work-family conflict (Mauno et al., 2013).
		• Positive relationships of job autonomy, skill variety, and social support with job satisfaction are weaker among older compared to younger workers (Besen et al., 2013).
		• Positive relationship of job autonomy with job satisfaction is weaker among older compared to younger workers (Ng and Feldman, 2015).
		• Perceived discrimination at work impacts on older workers' job satisfaction more negatively than on younger workers' job satisfaction (Taylor et al., 2013).
	Other positive aspects of work attachment	• Relationships between job characteristics and work engagement are more influenced by the interaction between chronological age and its work-related covariates (i.e., job tenure, position type) than by chronological age *per se*. High-tenure workers (regardless of age) display a stronger drop in engagement than low-tenure workers when demands are high. Older workers seem to value job control more than younger workers (Ramos et al., 2016).
		• Positive relationships of job autonomy with affective organizational commitment and work engagement are weaker among older compared to younger workers (Ng and Feldman, 2015).
	Perceived job stress and other job strain indicators	• Demand-control model applies differently to older and younger workers. Specifically, the interaction effects of demand and control variables on perceived work stress are more prevalent and numerous for older workers than for younger workers. Among older workers, the availability of sufficient time to complete tasks and job autonomy buffer the positive relationship between deadlines and strain, and scheduling flexibility buffers the positive relationship between problem solving demands and strain (Shultz et al., 2010).
		• Job stressors are more strongly positively, and social support is more strongly negatively related to perceptions of work-family conflict among older workers than among younger workers (Matthews et al., 2010).
		• Negative relationship between task variety and burnout is stronger for younger compared to older workers, whereas the negative relationship between skill variety and turnover intentions is stronger for older compared to younger workers (Zaniboni et al., 2013).
		• Negative relationship between job autonomy and emotional exhaustion is stronger among older compared to younger workers, whereas the negative relationships between job autonomy, poor mental health, and job stress are weaker among older compared to younger workers (Ng and Feldman, 2015).
		• Interactions among job characteristics, age, and age covariates and their relationship with work-related health outcomes. High-tenure workers (regardless of age) display a stronger rise in burnout than low-tenure workers when demands are high (Ramos et al., 2016).
		• Among older workers, the availability of sufficient time to complete tasks and job autonomy buffers the positive relationship between deadlines and strain, and scheduling flexibility buffers the positive relationship between problem solving demands and strain (Besen et al., 2015).
**WORK CHARACTERISTICS AS MEDIATORS**		
• Job congruence	Job satisfaction	• Job congruence (i.e., match between workers' job-related needs and supplies) and work-related locus of control mediate the age-job satisfaction relationship; older workers are more satisfied with their work characteristics because their jobs better meet their age-related needs and because they feel that they can determine what happens to them in their job (White and Spector, 1987).
• Perceived time pressure and coworker support	Job satisfaction	• Perceived time pressure and coworker support mediate the curvilinear relationship between age and job satisfaction. Time pressure and coworker support are higher among workers in mid-career (Zacher et al., 2014b).
	Emotional exhaustion	• Perceived time pressure and coworker support mediate the curvilinear relationship between age and emotional exhaustion. Time pressure and coworker support are higher among workers in mid-career (Zacher et al., 2014b).

### Literature search strategy

We searched the comprehensive Google Scholar database from 1900 to July 2016 for research on age, work characteristics, and occupational well-being, using combinations and variations of the following keywords: job/work characteristics, health, well-being, age, aging, younger/older workers, moderation, moderator, interaction, mediation, mediator.

### Interaction effects of work characteristics and age on occupational well-being

We identified 16 studies that examined interaction effects of work characteristics and age on occupational well-being (see Figure 1, Pathway A). These studies examined both work characteristics and age as moderators of the respective other variable's relationships with occupational well-being indicators (for an overview of findings and specific patterns of interaction effects found, see Table 1). Seven studies focused on job satisfaction as outcome variable (Riordan et al., 2003; Besen et al., 2013; Bos et al., 2013; Krumm et al., 2013; Mauno et al., 2013; Taylor et al., 2013; Zaniboni et al., 2016), or on outcome variables that have been shown to be positively related to job satisfaction (cf. Zacher and Yang, 2016), including perceptions of future work opportunities (Zacher and Frese, 2009, 2011; Zacher et al., 2010) and work engagement (Ramos et al., 2016). In addition to job satisfaction, six studies found interaction effects of work characteristics and age on indicators of perceived job stress and strain (Matthews et al., 2010; Shultz et al., 2010; Zaniboni et al., 2013; Besen et al., 2015; Hertel et al., 2015; Ramos et al., 2016). As can be seen in Table 1, the patterns of interaction effects of work characteristics and age on occupational well-being are diverse and complex; it appears that the interaction patterns depend not only on the specific work characteristics, but also on the specific occupational well-being indicators under consideration.

In addition to the primary studies, a recent meta-analysis examined age as a moderator of relationships between job autonomy and different positive and negative indicators of occupational well-being (Ng and Feldman, 2015). Findings differed for the specific indicators of occupational well-being under consideration. The negative relationship between job autonomy and emotional exhaustion was stronger among older compared to younger workers, whereas the negative relationships of job autonomy with poor mental health and perceived job stress were weaker among older compared to younger workers. The positive relationships of job autonomy with job satisfaction, affective organizational commitment, and work engagement were also weaker among older compared to younger workers.

### Work characteristics as mediators of associations between age and occupational well-being

We identified only two studies that investigated work characteristics as mediators of associations between age and occupational well-being (Pathway B in Figure 1; White and Spector, 1987; Zacher et al., 2014b; see Table 1). While White and Spector (1987) examined work characteristics as mediators of the linear and positive relationship between age and job satisfaction, Zacher et al. (2014b) found that two work characteristics, time pressure and coworker support, mediated the curvilinear relationships of age with job satisfaction and emotional exhaustion. The latter findings are consistent with research suggesting that the bivariate relationship between age and occupational well-being is better characterized by a U-shaped pattern, with younger, and older workers experiencing greater well-being than workers in mid-career (Birdi et al., 1995; Clark et al., 1996; Hochwarter et al., 2001).

The lack of studies on work characteristics as mediators of the age-occupational well-being relationship is surprising, given that much evidence exists for significant bivariate relationships between age and occupational well-being indicators. More specifically, a number of meta-analyses found that age is negatively related to objective indicators of physical health, and positively related to subjective measures of occupational well-being. Regarding physical health, a meta-analysis by Ng and Feldman (2013) showed that age was positively related to indicators of ill-health such as blood pressure, cholesterol level, body mass index, insomnia, and muscle pain, but not meaningfully related to self-reported physical health, somatic and psychosomatic complaints, depression, and anxiety. Moreover, age was weakly negatively related to indicators of psychological strain, including fatigue, negative mood, anger, and irritation. In contrast, another meta-analysis found no relationship between age and irritation (Rauschenbach et al., 2013). Finally, a meta-analysis by Ng and Feldman (2010) showed higher levels of job satisfaction, organizational commitment, intrinsic work motivation, and perceived person-job fit, as well as lower levels of role ambiguity, role conflict, role overload, and emotional exhaustion among older workers compared to younger workers.

## Implications for future research

The majority of relevant theoretical frameworks, including the lifespan perspective on work design, person-environment interaction and fit theories, and models of successful aging at work, focus on interaction effects of work characteristics and age on occupational well-being, whereas only role theory has been used to explain the role of work characteristics as mediators of associations between age and occupational well-being indicators. Accordingly, our review showed that more empirical research has been conducted to date on interaction effects between work characteristics and age, whereas very few studies exist on work characteristics as mediators. Future work could combine role theory (Biddle, 1986) and research on different layers of context surrounding younger, mid-career, and older workers (Farr and Ringseis, 2002), to gain a better understanding of how work characteristics may change across the working life span and predict occupational well-being. Researchers could also examine a broader range of potentially age-related work characteristics, such as problem solving and information processing demands (Sparks et al., 2001; Kanfer and Ackerman, 2004; Morgeson and Humphrey, 2006). Importantly, both streams of research (i.e., interaction effects of work characteristics and age, work characteristics as mediators) should examine potential curvilinear relationships of age with work characteristics and occupational well-being, as research based on role theory suggests that mid-career workers have higher job demands, lower levels of coworker support, and subsequently, lower occupational well-being than younger and older workers (Zacher et al., 2014b). This research is important to inform theory and organizational practice regarding workers in the mid-career stage, which are often neglected in research on work and aging.

Future research on age, work characteristics, and occupational well-being also needs to overcome a number of methodological challenges. First, most research in this area is based on cross-sectional designs, which do not allow conclusions about age-related changes (i.e., aging), disentangling aging from cohort effects, or drawing conclusions about causal effects of work characteristics on occupational well-being. Future research should increasingly use longitudinal, cohort-sequential, as well as experimental and intervention designs. These designs can show how work characteristics and occupational well-being change over time and across workers' careers, and they can provide more rigorous evidence regarding the effects of work characteristics on younger and older workers' occupational well-being. Second, researchers in this area of research have relied heavily on subjective assessments of work characteristics. This raises the questions whether there are differences between age-related changes in actual work characteristics and age-related changes in perceptions of work characteristics, how these differences can be explained and, more generally, what drives these age-related changes. The subjective assessment of work characteristics will continue to be important, as workers' perceptions of their work characteristics are important proximal predictors of occupational well-being. In addition to perceived work characteristics, however, researchers could assess more objective work characteristics, for instance by obtaining expert ratings or archival data based on job analysis, to examine how age is related to these more distal predictors of occupational well-being.

Third, most research has focused on investigating two-way interactions between work characteristics and age or a few selected mediators of associations between age and occupational well-being. As relationships between age and occupational well-being outcomes are complex, future research should propose and test more comprehensive mediated moderation models based on the theoretical frameworks described in this article. Specifically, these models could propose mediators of the moderating effects of age on associations between work characteristics and occupational well-being. For instance, age-related differences in work experience and occupational future time perspective may explain why age interacts with work characteristics in predicting occupational well-being indicators. Moreover, more complex theoretical models could also include the mechanisms that may explain why some objective and perceived work characteristics change with age, and also why work characteristics result in occupational well-being outcomes (e.g., increase in motivation, goal striving). Finally, more complex theoretical models could include hypotheses on three-way interaction effects of worker age, person characteristics, and work characteristics on occupational well-being indicators. For instance, Zacher and Frese (2011) showed that the use of self-management strategies (selection, optimization, and compensation; Baltes and Baltes, 1990) was particularly important for older workers' perceptions of future work opportunities when job complexity was low.

## Conclusion

There is an increasing awareness in the organizational psychology literature that temporal factors such as worker age (Truxillo and Zaniboni, 2015) and career development (Fried et al., 2007) play a role in relationships between work characteristics and occupational well-being. In this article, we reviewed this emerging line of research. Several studies found that work characteristics interact with worker age in predicting indicators of occupational well-being, including important outcomes such as job satisfaction, work engagement, and emotional exhaustion. In contrast, only few studies found that work characteristics mediate linear and curvilinear relationships between age and occupational well-being indicators, despite evidence for significant bivariate associations between age and occupational well-being indicators. Further, research should use more sophisticated research designs to gain a better understanding of the role of age in associations between work characteristics and occupational well-being.

## Author contributions

HZ wrote the first version of the manuscript and AS edited and provided feedback on subsequent versions of the manuscript.

### Conflict of interest statement

The authors declare that the research was conducted in the absence of any commercial or financial relationships that could be construed as a potential conflict of interest.

## References

[B1] Ashforth B. E. (2001). Role Transitions in Organizational Life: An Identity-Based Perspective. Mahwah, NJ: Erlbaum.

[B2] Baltes P. B. (1987). Theoretical propositions of life-span developmental psychology: on the dynamics between growth and decline. Dev. Psychol. 23, 611–626. 10.1037/0012-1649.23.5.611

[B3] Baltes P. B. Baltes M. M. (1990). Psychological perspectives on successful aging: the model of selective optimization with compensation, in Successful Aging: Perspectives from the Behavioral Sciences, eds Baltes P. B. Baltes M. M. (New York, NY: Cambridge University Press), 1–34.

[B4] Baltes P. B. Lindenberger U. Staudinger U. M. (2006). Lifespan theory in developmental psychology, in Handbook of Child Psychology: Theoretical Models of Human Development, Vol. 1, 6th Edn., eds Damon W. Lerner R. M. (New York, NY: Wiley), 569–664.

[B5] Besen E. Matz-Costa C. Brown M. Smyer M. A. Pitt-Catsouphes M. (2013). Job characteristics, core self-evaluations, and job satisfaction: what's age got to do with it? Int. J. Aging Hum. Dev. 76, 269–295. 10.2190/AG.76.4.a 23855183

[B6] Besen E. Matz-Costa C. James J. B. Pitt-Catsouphes M. (2015). Factors buffering against the effects of job demands: how does age matter? J. Appl. Gerontol. 34, 73–101. 10.1177/0733464812460430 25548089

[B7] Biddle B. J. (1986). Recent development in role theory. Annu. Rev. Sociol. 12, 67–92. 10.1146/annurev.so.12.080186.000435

[B8] Birdi K. Warr P. B. Oswald A. (1995). Age differences in three components of employee well-being. Appl. Psychol. 44, 345–373. 10.1111/j.1464-0597.1995.tb01085.x

[B9] Bos J. T. Donders N. C. G. M. Schouteten R. L. J. Van der Gulden J. W. J. (2013). Age as a moderator in the relationship between work-related characteristics, job dissatisfaction and need for recovery. Ergonomics 56, 992–1005. 10.1080/00140139.2013.789553 23651411

[B10] Carstensen L. L. Isaacowitz D. M. Charles S. T. (1999). Taking time seriously: a theory of socioemotional selectivity. Am. Psychol. 54, 165–181. 10.1037/0003-066X.54.3.165 10199217

[B11] Charles S. T. Carstensen L. L. (2010). Social and emotional aging. Annu. Rev. Psychol. 61, 383–409. 10.1146/annurev.psych.093008.100448 19575618 PMC3950961

[B12] Clark A. Oswald A. Warr P. (1996). Is job satisfaction U-shaped in age? J. Occup. Organ. Psychol. 69, 57–81. 10.1111/j.2044-8325.1996.tb00600.x

[B13] Edwards J. R. Caplan R. D. Harrison R. V. (1998). Person-environment fit theory: conceptual foundations, empirical evidence, and directions for future research, in Theories of Organizational Stress, ed Cooper C. L. (Oxford, UK: Oxford University Press), 28–67.

[B14] Fairchild A. J. MacKinnon D. P. (2009). A general model for testing mediation and moderation effects. Prev. Sci. 10, 87–99. 10.1007/s11121-008-0109-6 19003535 PMC2908713

[B15] Farr J. L. Ringseis E. L. (2002). The older worker in organizational context: beyond the individual, in International Review of Industrial and Organizational Psychology, eds Cooper C. L. Robertson I. T. (Chichester, UK: Wiley), 31–75.

[B16] Feldman D. C. Vogel R. M. (2009). The aging process and person-environment fit, in Research in Careers, eds Baugh S. G. Sullivan S. E. (Charlotte, NC: Information Age Press), 1–25.

[B17] Finkelstein L. M. Truxillo D. Fraccaroli F. Kanfer R. (eds.). (2015). Facing the Challenges of a Multi-Age Workforce: A Use-Inspired Approach. New York, NY: Routledge.

[B18] Fried Y. Grant A. M. Levi A. S. Hadani M. Slowik L. H. (2007). Job design in temporal context: a career dynamics perspective. J. Organ. Behav. 28, 911–927. 10.1002/job.486

[B19] Griffiths A. (1999). Work design and management: the older worker. Exp. Aging Res. 25, 411–420. 10.1080/036107399243887 10553525

[B20] Hackman J. R. Oldham G. R. (1976). Motivation through the design of work: test of a theory. Organ. Behav. Hum. Perform. 16, 250–279. 10.1016/0030-5073(76)90016-7

[B21] Hertel G. Rauschenbach C. Thielgen M. Krumm S. (2015). Are older workers more active copers? Longitudinal effects of age-contingent coping on on strain at work. J. Organ. Behav. 36, 514–537. 10.1002/job.1995

[B22] Hertel G. Zacher H. (in press). Managing the aging workforce, in Handbook of Industrial, Work, & Organizational Psychology, eds Anderson N. Ones D. S. Viswesvaran C. Sinangil H. K. (New York, NY: Sage).

[B23] Hochwarter W. A. Ferris G. R. Perrewe P. L. Witt L. A. Kiewitz C. (2001). A note on the nonlinearity of the age-job-satisfaction relationship. J. Appl. Soc. Psychol. 31, 1223–1237. 10.1111/j.1559-1816.2001.tb02671.x

[B24] Huffman A. Culbertson S. S. Henning J. B. Goh A. (2013). Work-family conflict across the lifespan. J. Manage. Psychol. 28, 761–780. 10.1108/JMP-07-2013-0220

[B25] Kanfer R. Ackerman P. L. (2004). Aging, adult development, and work motivation. Acad. Manage. Rev. 29, 440–458. 10.5465/AMR.2004.13670969

[B26] Kooij D. T. A. M. (2015). Successful aging at work: the active role of employees. Work Aging Retirement 1, 309–319. 10.1093/workar/wav018

[B27] Kooij D. T. A. M. De Lange A. H. Jansen P. G. W. Dikkers J. S. E. (2008). Older workers' motivation to continue to work: five meanings of age. J. Manage. Psychol. 23, 364–394. 10.1108/02683940810869015

[B28] Krumm S. Grube A. Hertel G. (2013). No time for compromises: age as a moderator of the relation between needs–supply fit and job satisfaction. Eur. J. Work Organ. Psychol. 22, 547–562. 10.1080/1359432X.2012.676248

[B29] Lazarus R. S. Folkman S. (1984). Stress, Appraisal, and Coping. New York, NY: Springer.

[B30] Matthews K. (2015). Stress and well-being: Its relationship to work and retirement for older workers, in Encyclopedia of Geropsychology, ed Pachana N. A. (New York, NY: Springer).

[B31] Matthews R. A. Bulger C. A. Barnes-Farrell J. L. (2010). Work social supports, role stressors, and work–family conflict: the moderating effect of age. J. Vocat. Behav. 76, 78–90. 10.1016/j.jvb.2009.06.011

[B32] Mauno S. Ruokolainen M. Kinnunen U. (2013). Does aging make employees more resilient to job stress? Age as a moderator in the job stressor–well-being relationship in three Finnish occupational samples. Aging Ment. Health 17, 411–422. 10.1080/13607863.2012.747077 23215801

[B33] Morgeson F. P. Humphrey S. E. (2006). The Work Design Questionnaire (WDQ): developing and validating a comprehensive measure for assessing job design and the nature of work. J. Appl. Psychol. 91, 1321–1339. 10.1037/0021-9010.91.6.1321 17100487

[B34] Ng T. W. H. Feldman D. C. (2010). The relationship of age with job attitudes: a meta-analysis. Pers. Psychol. 63, 667–718. 10.1111/j.1744-6570.2010.01184.x

[B35] Ng T. W. H. Feldman D. C. (2013). Employee age and health. J. Vocat. Behav. 83, 336–345. 10.1016/j.jvb.2013.06.004

[B36] Ng T. W. H. Feldman D. C. (2015). The moderating effects of age in the relationships of job autonomy to work outcomes. Work Aging Retirement 1, 64–78. 10.1093/workar/wau003

[B37] Perry E. L. Dokko G. Golom F. D. (2012). The aging worker and person–environment fit, in The Oxford Handbook of Work and Aging, eds Hedge J. W. Borman W. C. (New York, NY: Oxford University Press), 187–212.

[B38] Ramos R. Jenny G. Bauer G. (2016). Age-related effects of job characteristics on burnout and work engagement. Occup. Med. 66, 230–237. 10.1093/occmed/kqv172 26810576

[B39] Rauschenbach C. Krumm S. Thielgen M. M. Hertel G. (2013). Age and work-related stress: a review and meta-analysis. J. Manage. Psychol. 28, 781–804. 10.1108/JMP-07-2013-0251

[B40] Riordan C. M. Griffith R. W. Weatherly E. W. (2003). Age and work-related outcomes: the moderating effects of status characteristics. J. Appl. Soc. Psychol. 33, 37–57. 10.1111/j.1559-1816.2003.tb02072.x

[B41] Salthouse T. A. (2006). Mental exercise and mental aging: evaluating the validity of the “use it or lose it” hypothesis. Persp. Psychol. Sci. 1, 68–87. 10.1111/j.1745-6916.2006.00005.x 26151186

[B42] Scheibe S. Zacher H. (2013). A lifespan perspective on emotion regulation, stress, and well-being in the workplace, in Research in Occupational Stress and Well-Being, eds Perrewé P. L. Halbesleben J. Rosen C. C. (Bingley, UK: Emerald), 167–197.

[B43] Schmitt A. (in press). Occupational health, in The SAGE Encyclopedia of Lifespan Human Development, ed Bornstein M. H. (New York, NY: Sage).

[B44] Schmitt A. Bathen M. (in press). Occupational health, well-being, aging, in Encyclopedia of Geropsychology, ed Pachana N. A. (New York, NY: Springer).

[B45] Shultz K. S. Wang M. Crimmins E. M. Fisher G. G. (2010). Age differences in the demand-control model of work stress: an examination of data from 15 European countries. J. Appl. Gerontol. 29, 21–47. 10.1177/0733464809334286 20948986 PMC2952960

[B46] Sparks K. Faragher B. Cooper C. L. (2001). Well-being and occupational health in the 21st century workplace. J. Occup. Organ. Psychol. 74, 489–509. 10.1348/096317901167497

[B47] Taylor P. Mcloughlin C. Meyer D. Brooke E. (2013). Everyday discrimination in the workplace, job satisfaction and psychological wellbeing: age differences and moderating variables. Ageing Soc. 33, 1105–1138. 10.1017/S0144686X12000438

[B48] Truxillo D. M. Cadiz D. M. Hammer L. B. (2015). Supporting the aging workforce: a research review and recommendations for workplace intervention research. Annu. Rev. Organ. Psychol. Organ. Behav. 2, 351–381. 10.1146/annurev-orgpsych-032414-111435

[B49] Truxillo D. M. Cadiz D. M. Rineer J. R. Zaniboni S. Fraccaroli F. (2012). A lifespan perspective on job design: fitting the job and the worker to promote job satisfaction, engagement, and performance. Organ. Psychol. Rev. 2, 340–360. 10.1177/2041386612454043

[B50] Truxillo D. M. Zaniboni S. (2015). Work design and aging, in Encyclopedia of Geropsychology, ed Pachana N. A. (New York, NY: Springer).

[B51] Weiss H. M. Cropanzano R. (1996). Affective events theory: a theoretical discussion of the structure, causes and consequences of affective experiences at work, in Research in Organizational Behavior, eds Staw B. M. Cummings L. L. (Greenwich, CT: JAI Press), 1–74.

[B52] White A. T. Spector P. E. (1987). An investigation of age-related factors in the age-job satisfaction relationship. Psychol. Aging 2, 261–265. 10.1037/0882-7974.2.3.261 3268217

[B53] World Health Organization (2004). Promoting Mental Health: Concepts, Emerging Evidence, Practice (Summary Report). Geneva: Author.

[B54] Zacher H. (2015). Successful aging at work. Work Aging Retirement 1, 4–25. 10.1093/workar/wau006

[B55] Zacher H. Feldman D. C. Schulz H. (2014a). Age, occupational strain, and well-being: a person-environment fit perspective, in Research in Occupational Stress and Well-Being, eds Perrewé P. L. Halbesleben J. Rosen C. C. (Bingley, UK: Emerald), 83–111.

[B56] Zacher H. Frese M. (2009). Remaining time and opportunities at work: relationships between age, work characteristics, and occupational future time perspective. Psychol. Aging 24, 487–493. 10.1037/a0015425 19485664

[B57] Zacher H. Frese M. (2011). Maintaining a focus on opportunities at work: the interplay between age, job complexity, and the use of selection, optimization, and compensation strategies. J. Organ. Behav. 32, 291–318. 10.1002/job.683

[B58] Zacher H. Heusner S. Schmitz M. Zwierzanska M. M. Frese M. (2010). Focus on opportunities as a mediator of the relationships between age, job complexity, and work performance. J. Vocat. Behav. 76, 374–386. 10.1016/j.jvb.2009.09.001

[B59] Zacher H. Jimmieson N. L. Bordia P. (2014b). Time pressure and coworker support mediate the curvilinear relationship between age and occupational well-being. J. Occup. Health Psychol. 19, 462–475. 10.1037/a0036995 24885685

[B60] Zacher H. Yang J. (2016). Organizational climate for successful aging. Front. Psychol. 7, 1007. 10.3389/fpsyg.2016.01007 27458405 PMC4930930

[B61] Zaniboni S. Truxillo D. M. Fraccaroli F. (2013). Differential effects of task variety and skill variety on burnout and turnover intentions for older and younger workers. Eur. J. Work Organ. Psychol. 22, 306–317. 10.1080/1359432X.2013.782288

[B62] Zaniboni S. Truxillo D. M. Rineer J. R. Bodner T. E. Hammer L. B. Krainer M. (2016). Relating age, decision authority, job satisfaction, and mental health: a study of construction workers. Work Aging Retirement 2, 428–435. 10.1093/workar/waw006

